# Integrating transcriptomic and metabolomic analysis of hormone pathways in *Acer rubrum* during developmental leaf senescence

**DOI:** 10.1186/s12870-020-02628-5

**Published:** 2020-09-03

**Authors:** Chen Zhu, Lu Xiaoyu, Gao Junlan, Xuan Yun, Ren Jie

**Affiliations:** 1 Institute of Agricultural Engineering, Anhui Academy of Agricultural Sciences, 40 Nongkenanlu, Hefei, Anhui 230031 P.R. China; 2 College of Forestry and Landscape Architecture, Anhui Agricultural University, 130 Changjiangxilu, Hefei, Anhui 230036 P.R. China

**Keywords:** *Acer rubrum*, Transcriptome, Metabolomics, Hormone pathways, Leaf senescence

## Abstract

**Background:**

To fully elucidate the roles and mechanisms of plant hormones in leaf senescence, we adopted an integrated analysis of both non-senescing and senescing leaves from red maple with transcriptome and metabolome data.

**Results:**

Transcription and metabolite profiles were generated through a combination of deep sequencing, third-generation sequencing data analysis, and ultrahigh-performance liquid chromatograph Q extractive mass spectrometry (UHPLC-QE-MS), respectively. We investigated the accumulation of compounds and the expression of biosynthesis and signaling genes for eight hormones. The results revealed that ethylene and abscisic acid concentrations increased during the leaf senescence process, while the contents of cytokinin, auxin, jasmonic acid, and salicylic acid continued to decrease. Correlation tests between the hormone content and transcriptional changes were analyzed, and in six pathways, genes closely linked with leaf senescence were identified.

**Conclusions:**

These results will enrich our understanding of the mechanisms of plant hormones that regulate leaf senescence in red maple, while establishing a foundation for the genetic modification of *Acer* in the future.

## Background

The senescence of plant leaves is a proactive and genetically precise process that is regulated by a series of internal and external factors [1]. Many environmental stresses, such as extreme temperatures, drought, nutrient deficiencies, inadequate light/shadowing, or complete darkness, and biotic stress (e.g., bacterial infections) can induce senescence [2, 3]. Phytohormone levels are determined by plant age and stress during the induction and propagation of leaf senescence [4]. These internal and external factors may act alone or in combination. Among these, plant hormones are one class of organics that are generated in certain parts of plants, which can regulate almost all aspects of plant growth and development, while inducing physiological reactions at low concentrations [5]. Currently, research on the regulation and mechanisms of plant hormones in plant senescence has made clear progress [6].

According to the role of plant senescence, previous research has shown that, in general, phytohormones can be divided into two types: senescence promoters and retardants [7]. The former encompass ethylene (ET), abscisic acid (ABA), jasmonic acid (JA), salicylic acid (SA), and brassinosteroid (BR), whereas the latter include cytokinin (CK), auxin (IAA), and gibberellin (GA). A series of studies have confirmed that ethylene promotes senescence when the leaves reach a certain age or are mature enough [8]. Research has also shown that abscisic acid inhibits stomata closure during leaf senescence, which leads to significant water loss in leaves and their subsequent death. Simultaneously, it ensures that there is sufficient oxygen available in leaves for increased breathing during aging [9]. There has been considerable progress in the study of the role of jasmonic acid and salicylic acid in leaf senescence [10, 11]. The role of brassinosteroid in leaf senescence has been primarily addressed in studies of physiology and genetics. Following the treatment of leaves with brassinosteroid (24-Epibrassiolide; EBR), the malondialdehyde content of plants increases and reactive oxygen species is inhibited, which leads to leaf senescence [12]. However, its unique role in aging needs to be investigated in depth. In contrast, it has been verified that cytokinins have an inhibitory effect on leaf senescence [13]. The inhibitory effect of gibberellin on leaf senescence is not as obvious as for cytokinins [14]. Traditionally, auxin has been thought to inhibit plant senescence [15]. It is worthy of mention that these phytohormones do not work in isolation, and they are often employed in variable combinations to achieve the regulation of senescence [16, 17].

Leaf senescence is the final stage of the life history of leaves from maturity to demise, as they gradually yellow. This is accompanied by the transfer of nutrients from old leaves to new leaves and seeds [18]. Leaf senescence derives from the long-term evolution of plants, which assists them in adapting to environmental changes and maintaining efficient energy use [19]. However, the premature senescence of leaves seriously impacts photosynthesis time and nutrient transport in plants, which in turn reduces the yields and quality of crops [20]. The genus *Acer L*., also referred to as red maple, is among the most important tree and shrub genus in the northern hemisphere [21–25]. Maple trees are of high ornamental value and are attractive species for gardening. In addition to their outstanding landscape applications, many species of *Acer* also make contributions as major raw materials for maple sugar, maple syrup, furniture, and lumber [26]. Furthermore, a high number of maple phytochemicals have been found to possess potential antioxidant, antitumor, and anti-inflammatory activities [27–31]. Therefore, the study of leaf senescence not only has high scientific significance for exploring the life cycle and functional phenomena of this species, but also has important implications for red maple production.

The metabolism of plants is a networked relationship. The regulation of plant responses to environmental factors not only impacts individual metabolic pathways, but also influences the balance of the overall metabolic network [32]. Compared with traditional physiological studies and genetic phenotypic analyses, high-throughput omics research techniques can more systematically observe the physiological changes in plants and may have a stronger potential for discovering new genes [33, 34]. Recently, the association analyses of different omics platforms (transcriptomics and metabolomics) have gradually emerged as a new trend in the area of plant metabolism research [35–41]. For this study, we merged transcriptomics and metabolomics and studied eight common hormones in *Acer rubrum* (ethylene, abscisic acid, cytokinin, auxin, jasmonic acid, salicylic acid, brassinosteroid, and gibberellin). Changes in the levels of metabolites and expression levels of pathway genes in leaf senescence demonstrated the mechanisms through which different hormones regulate the process of senescence in plant leaves. This comprises the first ever complete transcriptomics and metabolomics analysis of the regulation of phytohormones involved in leaf senescence in *Acer rubrum*. The results of this study can enrich our understanding of the active mechanisms of phytohormones in *Acer.*

## Methods

### Plant materials and sample preparation

**Fig. 1:**
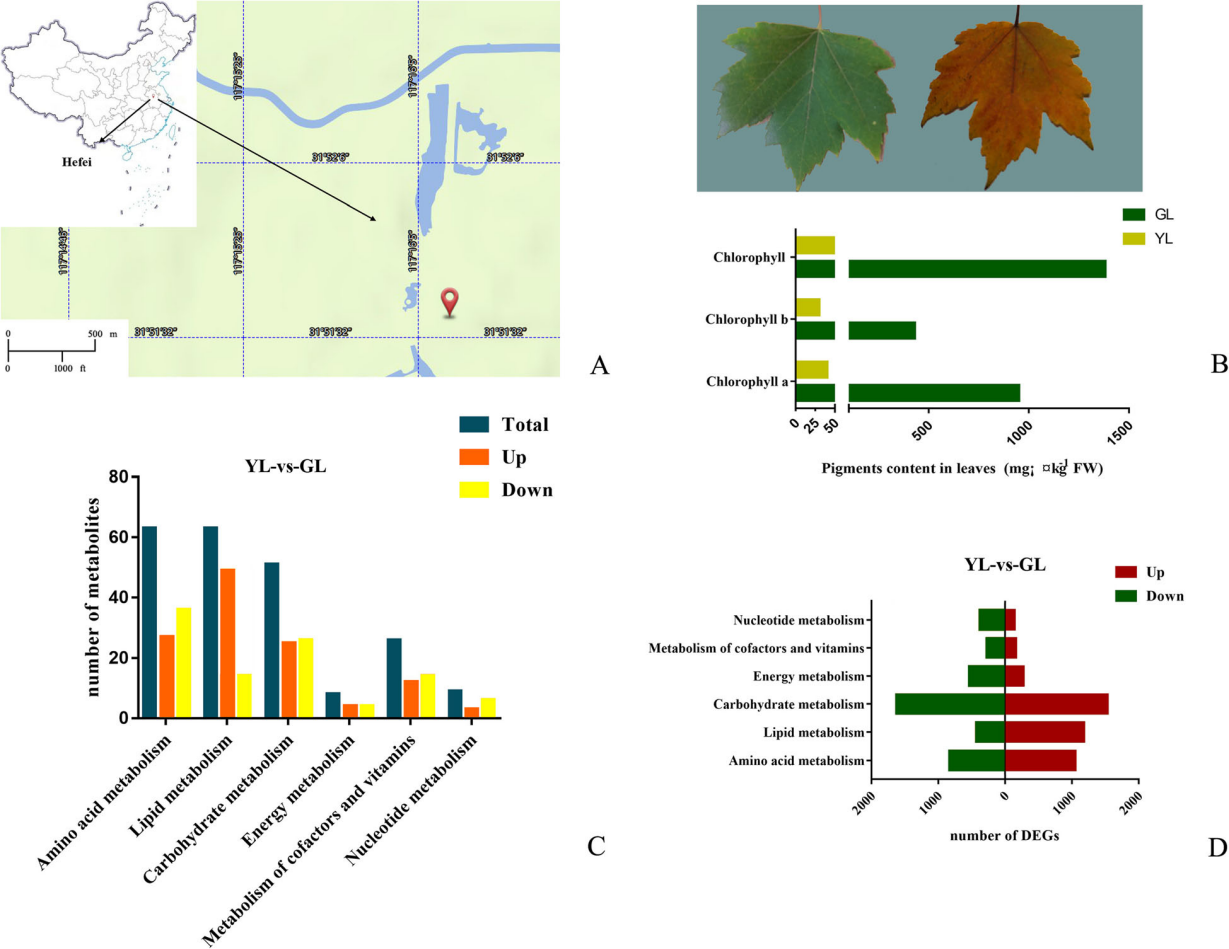
Leaf color and biochemical analysis of changes in red maple during leaf senescence. **a** Sampling location. The source of map depicted in (**a)** is from BIGMAP Company. **b** Stages of leaf senescence. GL, non-senescing leaves, fully expanded green leaves; YL, senescing leaves, 100% yellowing of the leaf surface; ~ 95% chlorophyll loss. **c** Changes in the relative populations of biological macromolecules during leaf senescence. “Total” refers to the total number of metabolites changed from senescent to non-senescent leaves. **d** Statistics of differential genes in macromolecular metabolic pathways during leaf senescence. “GL-vs-YL” means “non-senescing leaves compared senescing leaves”

Plants were planted in our test plots at the Anhui Academy of Agricultural Sciences, Hefei, China (Fig. 1a) (31.86 N latitude, 117.27 E longitude). Plant seeds from Canada Forest Service, presented by Dr. Shiyou Li. Non-senescing leaves (fully expanded green leaves) and senescing leaves (100% yellowing leaf surface, ~ 95% chlorophyll loss) were collected in middle-to-late November. Samples (20 leaves of *Acer rubrum* constituting one biologicalreplicate) for non-senescing leaves and senescing leaves were subjected to freeze drying, and then stored at − 80 °C in a deep freezer. The voucher specimens were deposited in Institute of Agricultural Engineering, Anhui Academy of Agricultural Sciences. Dr. Qian Zhu undertook the formal identification of the samples. This research examines a native plant species that does not require any special permit.

### Metabolic analysis

#### Metabolite extraction

We used a mixture comprising of 40% methanol (LC–MS grade, CNW Technologies): 40% acetonitrile (LC–MS grade, CNW Technologies): 20% water (v: v: v) as an extract solvent. The ratio was 0.05 g of leaves (each sample set consisted of six biological replicates) to 1 mL of solvent, and the solutions were vortexed for 30 s. The samples were homogenized (45 Hz, 4 min, JXFSTPRP-24; Jingxin Technology), and sonicated (5 min in ice-water bath). This step was repeated three times, after which the samples were left to stand at − 20 °C for 1 h, and underwent centrifugation (Heraeus Fresco17; Thermo Fisher Scientific) for 15 min at 12000 rpm at 4 °C. The supernatants were introduced to LC-MS vials for UHPLC-QE Orbitrap/MS detection. Equivalent amounts of supernatants from all samples were mixed as quality-control (QC) samples for testing.

#### LC-MS/MS analysis

LC-MS/MS analysis was conducted using a UHPLC system (1290; Agilent, Technologies) and Q Exactive Orbitrap (Thermo Fisher Scientific). Chromatographic separation was performed using a UPLC HSS T3 column (2.1 mm × 100 mm, 1.8 μm) [42].

The mobile phase was comprised of the following components:

- Positive mode: mobile phase A: formic acid (0.1%) in water solution; mobile phase B: acetonitrile.
- Negative mode: mobile phase A: ammonium acetate (5 mmol/L) in water solution; mobile phase B: acetonitrile.

The flow rate was maintained at 500 μL/min. The gradient parameters of t elution were set as follows: 0 min, 99% A, 1% B; 1 min, 99% A, 1% B; 8 min, 1% A, 99% B; 10 min, 1% A, 99% B; 10.1 min, 99% A, 1% B; 12 min, 99% A, 1% B. For the LC/MS experiments, QE mass spectrometer were able to perform MS/MS acquisition using information-dependent acquisition (IDA). In this mode, the acquisition software (Xcalibur 4.0.27, Thermo) continuously evaluates and triggers the acquisition of MS/MS spectra based on pre-selected criteria when acquiring full scan measurement MS data. Mass data acquisition was achieved in two ways: using ESI^+^ (electrospray ionization-positive ion mode) and ESI^−^ (electrospray ionization-negative ion mode). The following settings were used (sheath gas flow rate: 45 Arb, auxiliary gas flow rate: 15 Arb, capillary temperature:320 °C, full ms resolution:70,000, MS/MS resolution:17500, collision energy:20/40/60 eV, spray voltage:3.8kv (positive) or − 3.1kv (negative), respectively.

#### Data preprocessing and annotation

ProteoWizard software was employed to convert the raw MS data (.raw) to mzML format, after which the R package XCMS (version 3.2) was used to perform retention time correction, peak identification, peak extraction, peak integration, and peak alignment [43]. OSI-SMMS (version 1.0, Dalian Dashuo Information Technology Co., Ltd.) software was used in conjunction with an in-house MS/MS database for substance identification. Multivariate statistical analyses (PCA analysis, PLS-DA analysis, opls-da analysis) were carried out using the normalized data matrix. Differential metabolites were screened through the combination of multivariate statistical analysis opls-da and single variable statistical analysis (student’s t test), whereas metabolic pathway KEGG enrichment analysis was conducted using non-commercial databases (Supplementary Table S1).

### Combination sequencing analysis

#### RNA sequencing experiment

Transcriptome sequencing analyses were assessed as previously described [44]. A combination of next-generation sequencing (NGS) and single-molecule real-time (SMRT) sequencing were adopted for this study. The experiment involved total RNA isolation (using the RNAprep kit DP441; Tiangen,) followed by conversion to the Iso-Seq library of short cDNA fragments according to the Isoform Sequencing protocol (Iso-Seq) using the Clontech SMARTer PCR cDNA Synthesis Kit. Sequencing used the BluePippin Size Selection System protocol as described by Pacific Biosciences (PN 100–092–800-03).

#### KEGG enrichment and differential expression analysis

To capture the phytohormone-related genes, we utilized the KEGG (http://www.genome.jp/kegg/) online database for the pathway mapping of target genes. These included tryptophan metabolism (auxin synthesis pathway; pathway ID 00380), zeatin biosynthesis (cytokinin synthesis pathway; pathway ID 00908), diterpenoid biosynthesis (gibberellin synthesis pathway; pathway ID 00904), carotenoid biosynthesis (abscisic acid synthesis pathway; pathway ID 00906), cysteine and methionine metabolism (ethylene synthesis pathway; pathway ID 00270), brassinosteroid biosynthesis (brassinosteroid synthesis pathway; pathway ID 00905), alpha-Linolenic acid metabolism (jasmonic acid synthesis pathway; pathway ID 00592), phenylalanine metabolism (salicylic acid synthesis pathway; pathway ID 00360), plant hormone signal transduction (plant hormone signal transduction; pathway ID 04075). By comparing the values (|log2(FoldChange)| > 1 & *P* value < 0.01) of fragments per kilobase of transcript per million mapped reads (FPKM), the differentially expressed genes (DEGs) were selected. Heatmaps of these DEGs were generated using Rpackage (Supplementary Table S2).

#### Correlation analysis

The obtained metabolome and transcriptome data were applied to the calculation of correlation coefficients (Spearman’s rank correlation test, |R| > 0.8) by using GraphPad Prism (8.0) software. The metabolome and transcriptome relationships in red maple were visualized using Cytoscape (version 3.6.1) (Supplementary Table S3).

## Results

### Leaf senescence

Leaf senescence is the last stage in the life history of red maple leaves, from maturation tosenescence, where the most obvious external change is yellowing. Our previous research revealed that the yellowing of the senescing leaves of red maple is primarily attributed to the degradation of chlorophyll, not the emergence of yellow plant-based pigments such as carotenoids [44]. We used ultrahigh-performance liquid chromatograph Q extractive mass spectrometry (UHPLC-QE-MS) to analyze the metabolism of non-senescing leaves (fully expanded non-senescing leaves) and senescing leaves (100% yellowing of the leaf surface; ~ 95% chlorophyll loss) (Fig. 1b). The test results showed that there are many different metabolites in the metabolic pathways of amino acids, lipids, and other biological macromolecules in the non-senescing leaves compared to senescing leaves (Fig. 1c, Supplementary Table S4).

Leaf senescence is a genetically regulated recycling process in the lifecycle of plants in functional organs with photosynthesis to the degradation stage. To evaluate the leaf senescence process in *Acer rubrum*, we performed combination sequencing with non-senescing leaves and senescing leaves. Based on the sequencing results, we compared the numbers of differentially expressed genes in related pathways such as amino acid metabolism, lipid metabolism, carbohydrate metabolism, energy metabolism, the metabolism of cofactors and vitamins, and nucleotide metabolism in non-senescing and senescing leaves. For the comparison (non-senescing vs senescing leaves), the greatest number of DEGs were found in carbohydrate metabolism (1623 up- and 1530 down-regulated). Conversely, the metabolism of cofactors and vitamins represented the smallest group, with 269 up-and 163 down-regulated unigenes (Fig. 1d, Supplementary Table S5).

### Ethylene

**Fig. 2:**
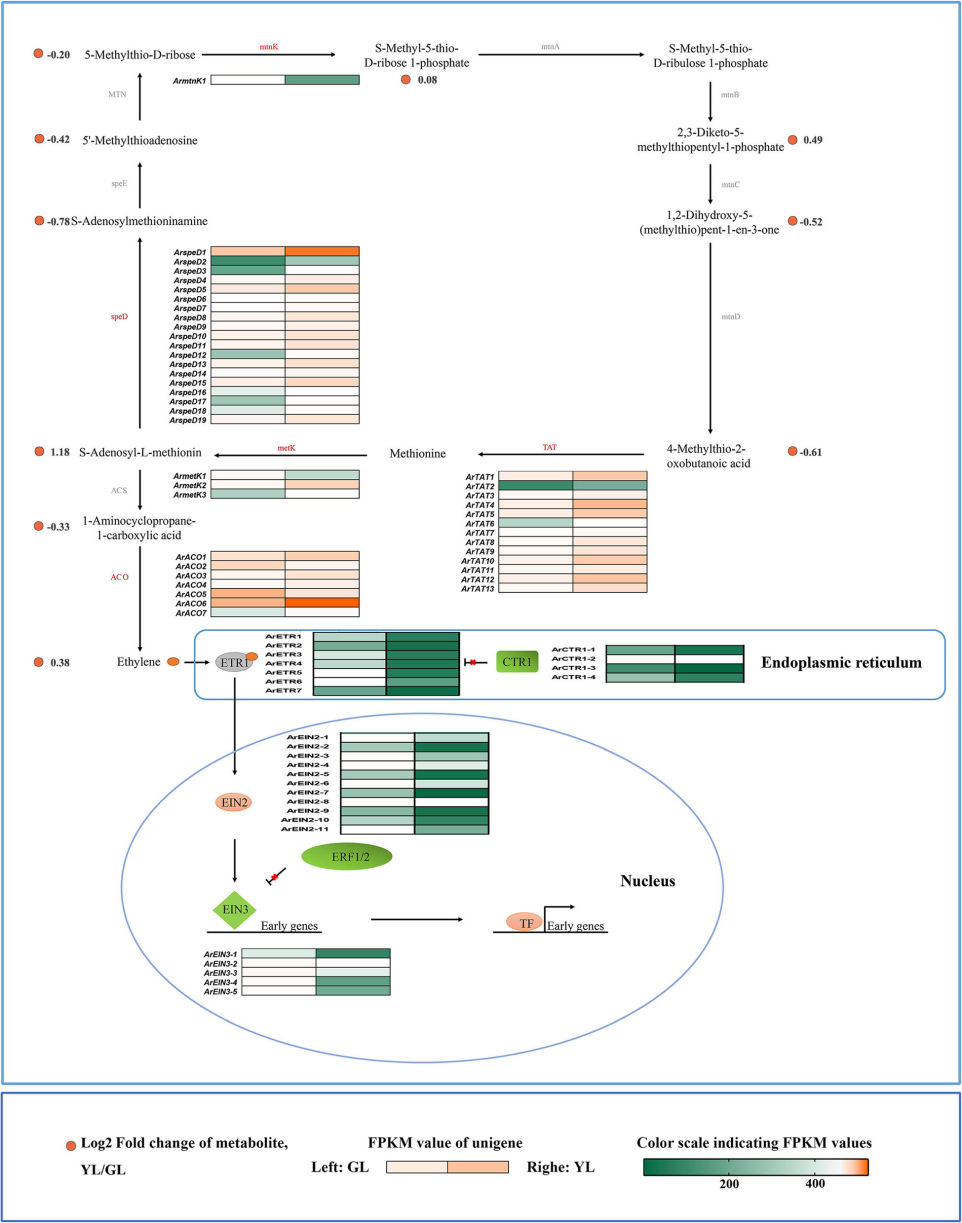
Biosynthetic and signal transduction pathways of ethylene. mtnK, 5-methylthioribose kinase; mtnA, Methylthioribose-1-phosphate isomerase; MTN, 5′-methylthioadenosine nucleosidase; mtnB, Methylthioribulose-1-phosphate dehydratase; speE, Spermidine synthase; mtnC, 2,3-diketo-5-methylthiopentyl-1-phosphate enolase-phosphatase; speD, S-adenosylmethionine decarboxylase proenzyme; mtnD, 1,2-dihydroxy-3-keto-5-methylthiopentene dioxygenase; metK, S-adenosylmethionine synthetase; TAT, tyrosine aminotransferase; ACS, 1-aminocyclopropane-1-carboxylate synthase; ACO, aminocyclopropane carboxylate oxidase; ETR, ethylene receptor; CTR, constitutive; EIN2, ethylene insensitive 2; EIN3, ethylene insensitive 3, ERF1/2, ethylene-responsive transcription factor 1/2; TF, transcription factor. GL, non-senescing leaves, fully expanded green leaves; YL, senescing leaves, completely yellow; ~ 95% chlorophyll loss. Colored cells represent the expression profiles of differentially expressed genes (DEGs). Heatmaps were generated with the Rpackage and the color bar at the lower right. Orange dots show the fold change of the content of compounds in non-senescing and senescing leaves

The phytohormone ethylene is extensively involved in many plant processes, where previous studies have found that ethylene levels were linked to leaf senescence. In many species, ethylene treatment can initiate alterations in aging characteristics including promoting the reduction of chlorophyll, chlorophyll–protein complexes, and other macromolecular substances, while increasing the activity of proteases and other metabolic enzymes [45, 46]. Considerable research has summarized the synthesis pathway of ethylene as the conversion of methionine to S-adenosylmethionine (SAM), SAM to 1-aminocyclopropane-1-carboxylic acid (ACC), and ACC to ethylene [47]. As there is alimited quantity of methionine in plants, the methylthio group, is recycled to replenish methionine levels and sustain ethylene biosynthesis. The detected ethylene and related compounds in the metabolic pathway are arranged in corresponding positions (Fig. 2). As displayed, S-Adenosyl-L-methionine, S-Methyl-5-thio-D-ribose 1-phosphate, 2,3-Diketo-5-methyl thiopentyl-1-phosphate, and ethylene were shown to be most abundant in senescing leaves, whereas S-Adenosylmethioninamine, 5′-Methylthioadenosine, 5-Methylthio-D-ribose, 1,2-Dihydroxy-5-(methylthio)pent-1-en-3-one, 4-methylthio-2-oxobutanoic acid, and 1-Aminocyclopropane-1-carboxylic acid were shown to be the more abundant in non-senescing leaves.

**Fig. 3:**
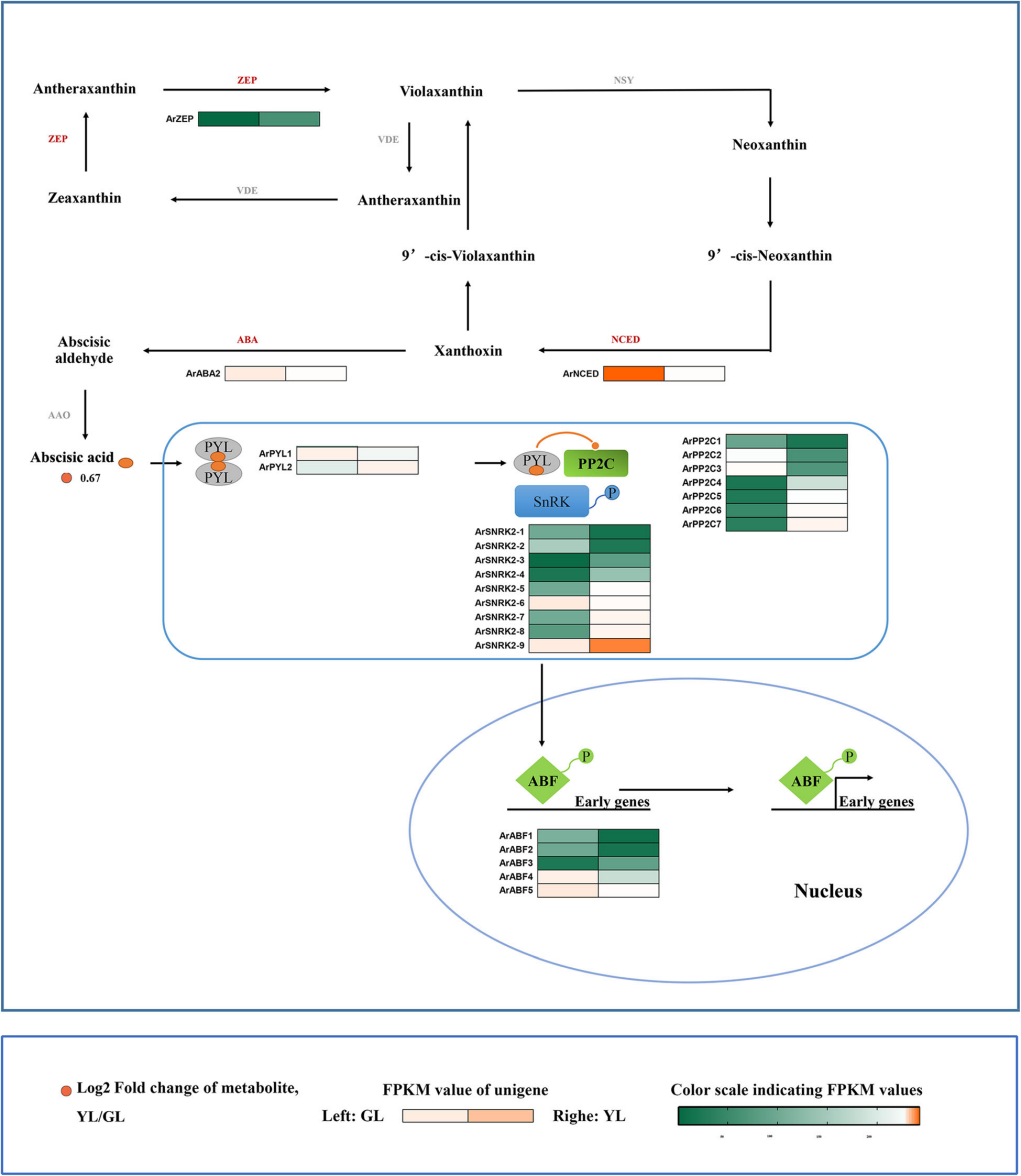
Biosynthetic and signal transduction pathways of abscisic acid. ZEP, zeaxanthin epoxidase; NSY, neoxanthin synthase; VDE, violaxanthin de-epoxidase; ABA, xanthoxin dehydrogenase; NCED, 9-cis-epoxycarotenoid dioxygenase; AAO, abscisic-aldehyde oxidase; PYL, pyrabactin-resistance 1-like; PP2C, protein phosphatase type 2C; SnRK, serine/threonine-protein kinase; ABF, abscisic acid responsive element binding factor. GL, non-senescing leaves, fully expanded green leaves; YL, senescing leaves, completely yellow; ~ 95% chlorophyll loss. The colored cells represent the expression profiles of differentially expressed genes (DEGs). The heatmaps were generated using the Rpackage and the color bar at the lower right. Orange dots show the fold change of the content of compounds in non-senescing and senescing leaves

Of the 43 differential expression genes (DEGs) involved in ethylene biosynthesis, 39 are highly expressed in senescing leaves (Fig. 3). ACC synthase (ACS) and ACC oxidase (ACO) are rate-limiting enzymes in the ethylene biosynthesis pathway. In our study, no detectable DEGs of ACS were observed. Seven DEGs of *ArACOs* were detected, where most (5/7) exhibited higher expression in senescing leaves. All of the 27 ET-signaling genes are up-regulated in non-senescing leaves.

### Abscisic acid

Abscisic acid (ABA) was once thought to be the most effective hormone in the promotion of leaf senescence in addition to ethylene. We investigated the enrichment of metabolites and genes involved in ABA synthesis, and the ABA-signaling pathway (Fig. 3). Our results showed that the content of abscisic acid was high in senescing leaves and exceeding that in non-senescing leaves. At the ABA synthesis stage, three genes demonstrated differential expression. Among them, *ArZEP* had a higher expression level in senescing leaves, while *ArNCED* and *ArABA2* were highly expressed in non-senescing leaves. Of the 23 ABA-signaling genes, 12 were up-regulated in senescing leaves (*ArPYL2, ArPP2C4/5/6/7, ArSNRK2–3/4/5/7/8/9, ArABF3*) and 11 were down-regulated (*ArPYL1, ArPP2C1/2/3, ArSNRK2–1/2/6, ArABF1/2/4/5*) (Fig. 3).

### Cytokinins

Cytokinins (CKs) are phytohormones, which play an important role in plant growth and development. The administration of exogenous cytokinins on ex vivo and living leaves can prevent leaf senescence [48]. By analyzing the levels of cytokinins in leaves prior to and following senescence, it was revealed that there was a negative correlation between cytokinins levels and aging processes in various tissues and plants [7, 49, 50]. Furthermore, genetic manipulation of cytokinin production in transgenic plants has provided very solid evidence for the inhibition role of cytokinins in leaf senescence [7, 51–53].

**Fig. 4:**
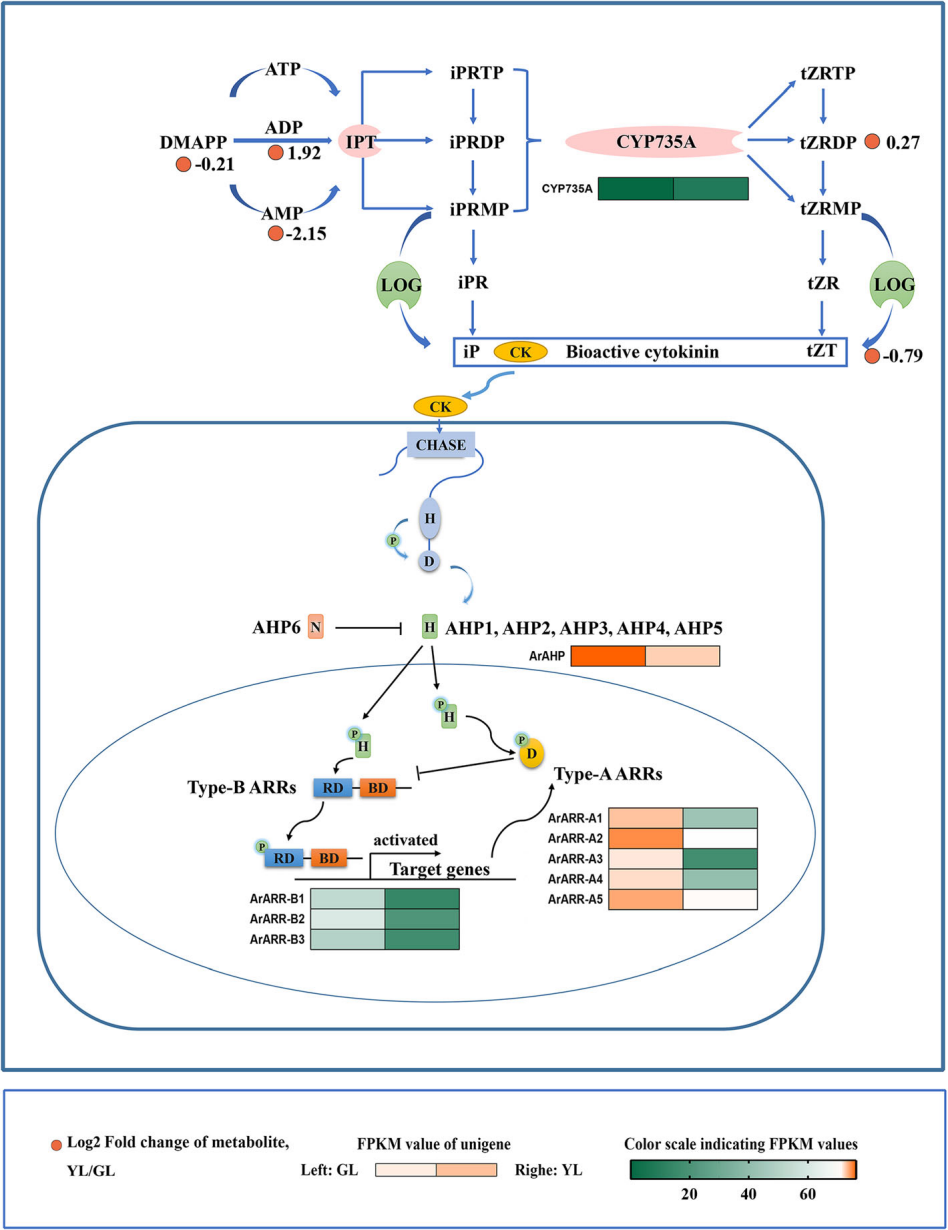
Biosynthetic and signal transduction pathways of cytokinins. DMAPP, dimethylallyl diphosphate; ATP, adenosine triphosphate; ADP, adenosine diphosphate; AMP, adenosine monophosphate; IPT, isopentenyl transferase; iPRTP, isopentenyl adenosine-5′-triphosphate; iPRDP, isopentenyl adenosine-5′-diphosphate; iPRMP, isopentenyl adenosine-5′-monophosphate; CYP735A, cytokinin trans-hydroxylase; tZRTP, trans-Zeatin riboside triphosphate; tZRDP, trans-Zeatin riboside diphosphate; tZRMP, trans-Zeatin riboside monophosphate; LOG, cytokinin-activating enzyme LONELY GUY; iPR, isopentenyl adenosine; tZR, trans-zeatin riboside; iP, isopentenyl adenine; tZT, trans-zeatin; AHP, histidine-containing phosphotransfer peotein; Type-A ARRs, type-A response regulators; Type-B ARRs, type-B response regulators. GL, non-senescing leaves, fully expanded green leaves; YL, senescing leaves, completely yellow; ~ 95% chlorophyll loss. The colored cell represents the expression profiles of differentially expressed genes (DEGs). The heatmaps were generated using the Rpackage and the color bar at the lower right. Orange dots show the fold change of the content of compounds in non-senescing and senescing leaves

In this study, the content of cytokinins in senescing leaves was far above that of non-senescing leaves, and genes related to various aspects of CK homeostasis were collected (Fig. 4). Cytokinin signaling is based on two-component system (TCS) that is achieved by the continuous transfer of phosphate groups between major components. Our results showedthat all differentially expressed genes in this signal transduction pathway were down-regulated in senescing leaves (Fig. 4).

### Auxin

Early study revealed that exogenous application of auxin was inversely related to leaf senescence, and that the endogenous auxin levels of many plants decreased with advancing age [54]. Plants achieve the synthesis of tryptophan to auxin through three pathways, named after the intermediate products, these are include the indole-3-pyruvate pathway, the tryptamine pathway, and the indole-3-acetonitrile pathway [55]. At present, these two of three synthetic pathways have not been fully investigated, and key genes controlling the relevant synthetic steps have yet to be discovered. Many of these steps are still potential models based on existing experiments.

**Fig. 5:**
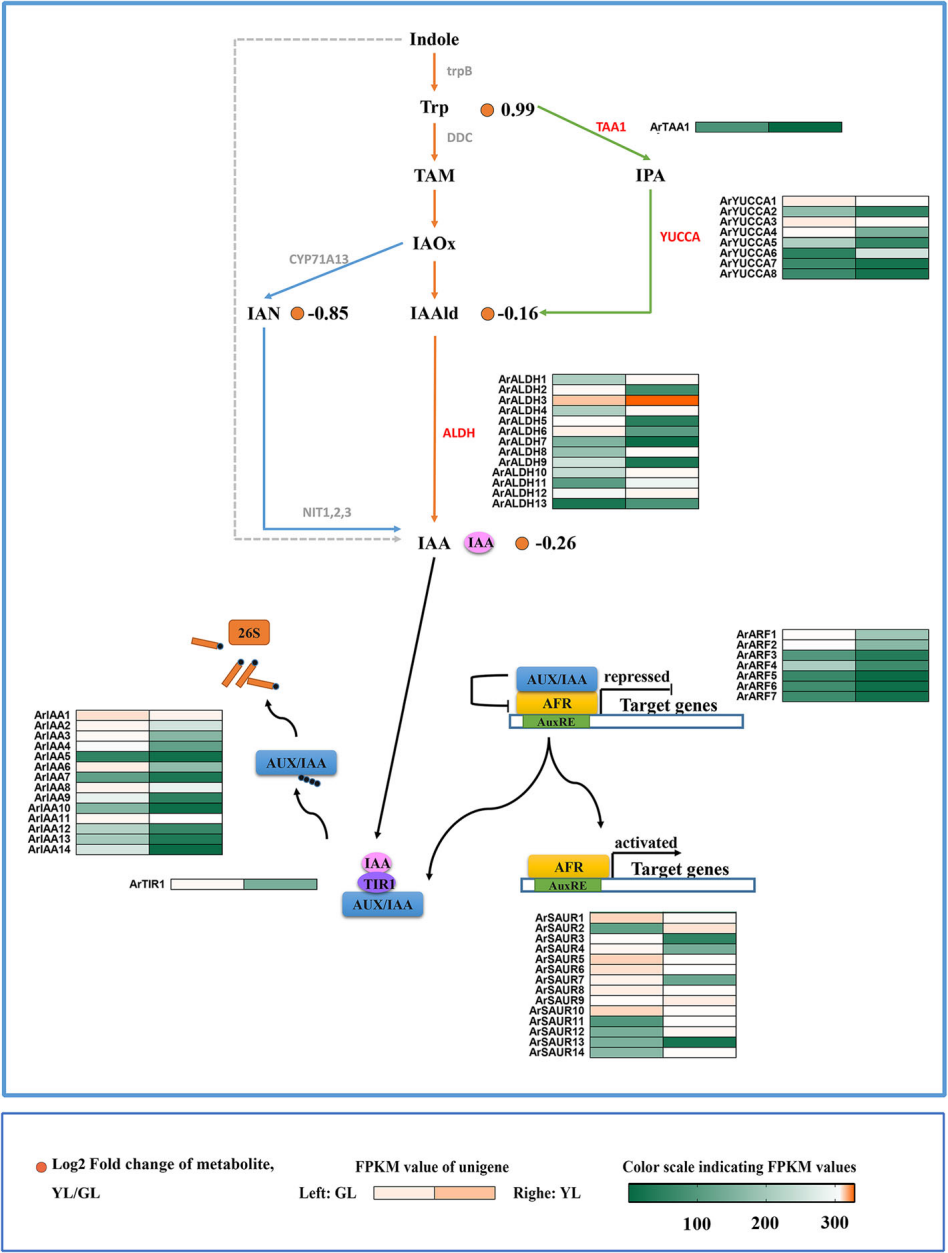
Biosynthetic and signal transduction pathways of auxin. trpB, tryptophan synthase beta chain; Trp, tryptophan; DDC, tryptophan decarboxylase; TAA, tryptophan aminotransferase; TAM, tryptamine; IPA, indole-3-pyruvate; CYP71A13, indoleacetaldoxime dehydratase; IAOx, indole 3-acetaldoxime; YUCCA, indole-3-pyruvate monooxygenase; IAAld, Indole-3-acetaldehyde; IAN, Indole-3-acetonitrile; ALDH, aldehyde dehydrogenase; NIT, nitrilase; IAA, Indole-3-acetate; AUX/IAA, auxin-responsive protein IAA; ARF, auxin response factor; TIR1, transport inhibitor response 1; SAUR, small auxin up-regulated RNA. GL, non-senescing leaves, fully expanded green leaves; YL, senescing leaves, completely yellow; almost 95% chlorophyll loss. The colored cells represent the expression profiles of differentially expressed genes (DEGs). Heatmaps were generated by Rpackage and the color bar at the lower right. Orange dots show the fold change of the content of compounds in non-senescing and senescing leaves

In senescing leaves of red maple, the tryptophan concentration is higher than in non-senescing leaves. Indole-3-acetaldehyde (IAAld), indole-3-acetonitrile (IAN), and indole-3-acetic acid (IAA) have a higher content in non-senescing leaves (Fig. 5). A total of 22 key differential expression genes were obtained in the auxin synthesis pathway, including the YUCCA, ALDH, and TAA1 family genes. There are seven YUCCA gene expression levels in senescing leaves that are significantly down-regulated (indole-3-pyruvate pathway). The indole-3-pyruvate pathway is the first completely elucidated auxin biosynthetic pathway, and the most fundamental and important auxin synthesis pathway in plants [56, 57].

Many studies have shown that YUCCA inactivation in this pathway reduces the synthesis of auxin in plants, whereas overexpression of the YUCCA gene leads to the overproduction of auxin, which causes developmental defects [58]. Therefore, YUCCA is a rate-limiting factor for auxin synthesis, and its gene expression pattern plays a crucial role in auxin synthesis. In this study, the expression of *ArYUCCA* genes in senescing leaves was down-regulated, suggesting its key role in auxin synthesis. Through differential gene screening, 35 differentially expressed genes, including TIR1, IAA, ARF, and SAUR family genes, were found in the auxin signal transduction pathway. The analysis of each family gene expression pattern revealed that the TIR1, IAA, and ARF gene families were up-regulated in non-senescing leaves, while the SAUR gene family had both up−/down-regulated genes in non-senescing leaves (Fig. 5).

### Jasmonic acid

**Fig. 6:**
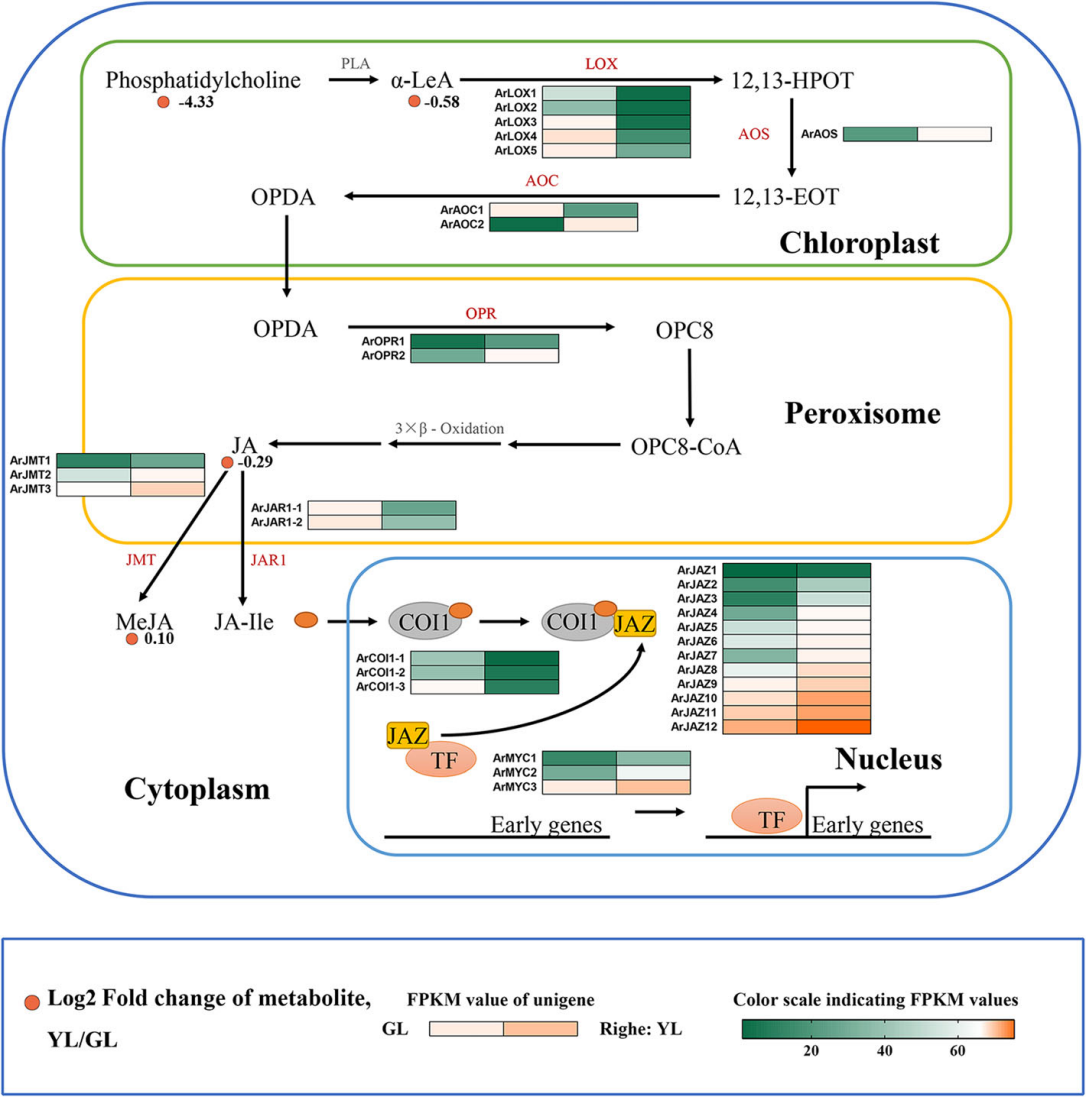
Biosynthetic and signal transduction pathways of jasmonic acid. LA, phospholipase A1; α-LeA, alpha-Linolenic acid; LOX, lipoxygenase; 12,13-HPOT, 12,13-hydroperoxylinoleic acid; AOS, allene oxide synthase; 12,13-EOT, 12,13-epoxyoctadecatrienoic acid; AOC, allene oxide cyclase; OPDA, oxophytodienoic acid; OPR, oxophytodienoic acid reductase; JA, jasmonic acid; JMT, jasmonic acid carboxyl methyltransferase; JAR1, jasmonic acid resistant 1; MeJA, Methyl jasmonate; JA-Ile, Jasmonoyl-L-isoleucine; COI1, coronatine-insensitive protein 1; JAZ, jasmonate ZIM domain-containing protein. GL, non-senescing leaves, fully expanded green leaves; YL, senescing leaves, completely yellow; ~ 95% chlorophyll loss. Colored cells represent the heatmaps of differentially expressed genes (DEGs). With the heatmaps, the redder the color, the higher the DEGs expression, and the greener the color, the lower the DEGs expression. Orange dots show the fold change the content of the compounds in non-senescing and senescing leaves

Jasmonic acid and its derivatives are an important class of plant hormone that is required for plant growth and development and assists with enduring stress and completing the life cycle. The first documented function of jasmonic acid was the promotion of senescence in isolated oat leaves [59, 60]. Therefore, we investigated the metabolite content and the expression patterns of DEGs in jasmonic acid biosynthesis and signal transduction related pathways of red maple (Fig. 6). The content of α-Linolenic acid and methyl jasmonate in non-senescing leaves was higher than that in senescing leaves, and the content of phosphatidylcholine and jasmonic acid was higher in the latter.

The expression of related genes showed a clear pattern, and the gene expression profiles of the same gene family, except for allene oxide cyclase (AOC), were consistent. Among them, one *ArAOC* gene and five lipoxygenase (LOX) genes were down-regulated in senescing leaves. Seven biosynthesis genes were up-regulated in senescing leaves, including allene oxide synthase (AOS), allene oxide cyclase (AOC), oxophytodienoate reductase (OPR), and jasmonate O-methyltransferase (JAT), which catalyzed a series of reactions in the JA-biosynthetic pathway. Jasmonate-resistant 1 (JAR1), coronatine insensitive 1 (COI1), jasmonate ZIM (JAZ) proteins, and the transcription factor MYC2 are important constituent elements of the JA-signaling pathway. The first two are down-regulated in senescing leaves, whereas the latter two are up-regulated.

### Salicylic acid

**Fig. 7:**
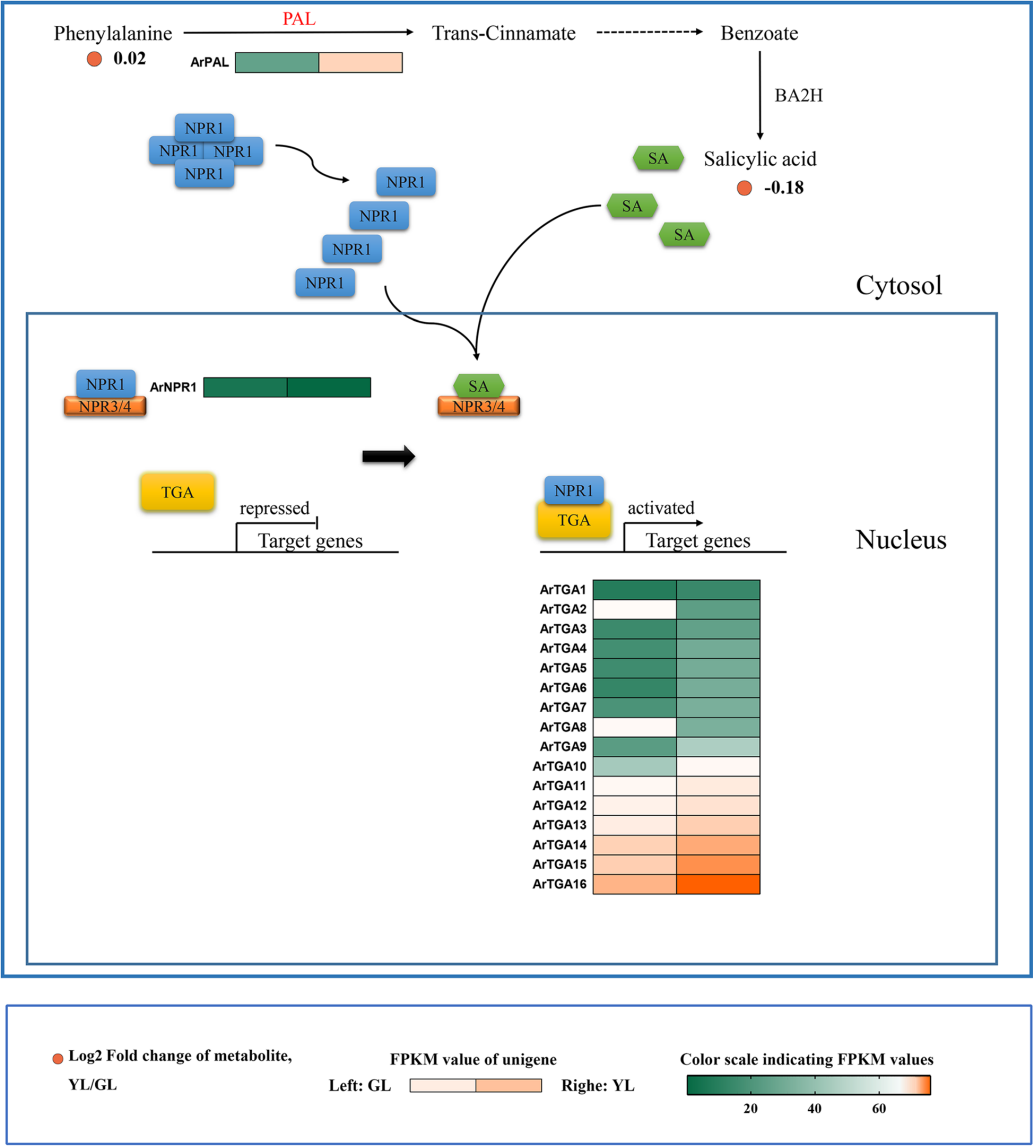
Biosynthetic and signal transduction pathways of salicylic acid. PAL, phenylalanine ammonia-lyase; BA2H, benzoate; NADPH: oxygen oxidoreductase (2-hydroxylating); NPR, nonexpressorofpathogenesis-relatedgenes1; TGA, transcription factor TGA. GL, non-senescing leaves, fully expanded green leaves; YL, senescing leaves, completely yellow; ~ 95% chlorophyll loss. Colored cells represent the expression profiles of differentially expressed genes (DEGs). Heatmaps were generated using the Rpackage and the color bar at the lower right. Orange dots show the fold change of the content of compounds in non-senescing and senescing leaves

Salicylic acid (SA) is a phenolic plant hormone that can regulate seed germination, cell growth, respiration, stomatal closure, stress response, nitrogen fixation, and the seed setting rate of various plants [61]. The role of salicylic acid in leaf senescence has also been recognized in recent years [62]. Therefore, we investigated the metabolite content and the expression of related genes in the salicylic acid SA synthesis, and the SA-signaling pathway in red maple (Fig. 7). The phenylalanine pathway was the first discovered salicylic acid synthesis pathway. We detected that the phenylalanine content was almost equivalent in both senescing and non-senescing leaves. However, the content of salicylic acid in non-senescing leaves of red maple is higher, although the expression of the core enzyme phenylalanine ammonia lyase (PAL) gene in the senescing leaves is much higher than that of non-senescing leaves. Interestingly, this result was different from those of other studies. In Arabidopsis, the content of endogenous salicylic acid increased with leaf senescence [63].

Studies have shown that salicylic acid signal transduction pathways are primarily NPR1-dependent pathways [64]. Following the depolymerization of NPR1 into a reactive monomer and transfer to the nucleus, it can interact directly with some members of the transcription factor TAG family to induce the expression of downstream disease resistance genes. The expression levels of *ArNPR1* gene in senescing leaves and non-senescing leaves were similar; however, 15 members of the 16 TGA genes were up regulated during senescence. Therefore, we hypothesized that the role of SA in the leaf senescence of red maple was dependent on the efficient expression of related genes in signal transduction pathways.

### Brassinosteroid

The plant hormone brassinosteroid (BR) play a role in a variety of developmental processes including leaf senescence. Some Arabidopsis mutants of BR biosynthesis (e.g., *det2*,) [65], or with the loss of a signal transduction pathway (e.g., *BRI1*) have delayed leaf senescence phenotypes and associated aging characteristics [66–69]. However, this phytohormone was not detected in our metabolome test results. Previous studies have shown that the content of brassinosteroid in plant tissues varies greatly [70]. Pollen, immature seeds, and fruits have the highest BR content. Young growing tissue contains higher BR levels; however, mature leaves have lower BR concentrations [71].

**Fig. 8:**
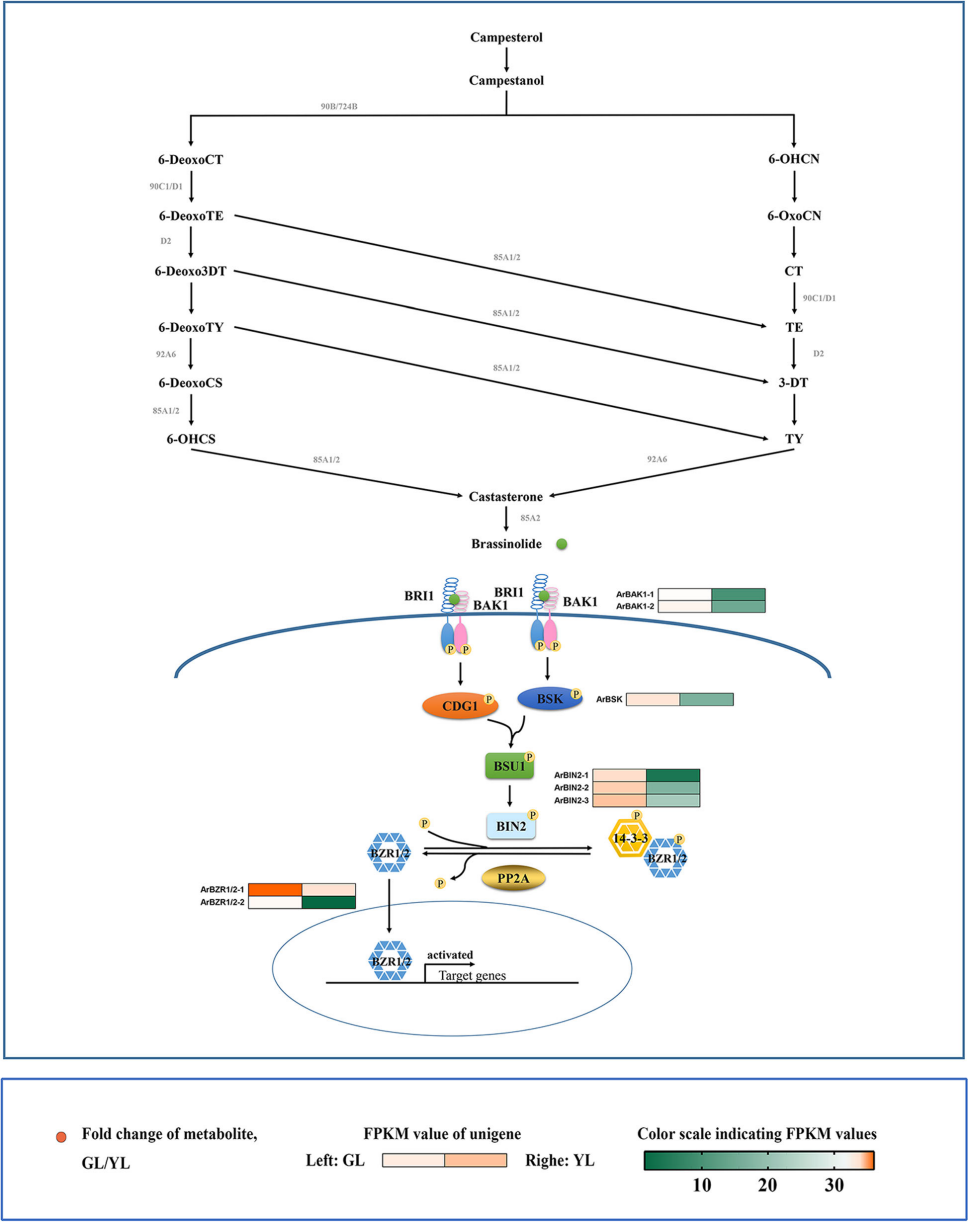
Biosynthetic and signal transduction pathways of brassinosteroids. 90B/724B, steroid 22-alpha-hydroxylase; 6-DeoxoCT, 6-Deoxocathasterone; 6-OHCN, 6alpha-Hydroxycampestanol; 6-DeoxoTE, 6-Deoxoteasterone; 6-OxoCN, 6-Oxocampestanol; 6-Deoxo3DT, 3-Dehydro-6-deoxoteasterone; CT, cathasterone; 6-DeoxoTY, 6-Deoxotyphasterol; TE, teasterone; 6-DeoxoCS, 6-Deoxocastasterone; 3-DT, 3-Dehydroteasterone; 6-OHCS, 6alpha-Hydroxy-castasterone; TY, Typhasterol; 85A1/2, brassinosteroid-6-oxidase 1; 92A6, typhasterol/6-deoxotyphasterol 2alpha-hydroxylase; 85A2, brassinosteroid-6-oxidase 2; BRI1, protein brassinosteroid insensitive 1; BAK1, brassinosteroid insensitive 1-associated receptor kinase 1; BSK, BR-signaling kinase; BSU1, serine/threonine-protein phosphatase; BIN2, brassinosteroid insensitive 2; BZR1/2, brassinosteroid resistant 1/2; PP2A, protein phosphatase type 2A. GL, non-senescing leaves, fully expanded green leaves; YL, senescing leaves, completely yellow; ~ 95% chlorophyll loss. Colored cells represent the expression profiles of differentially expressed genes (DEGs). Heatmaps were generated with the Rpackage and the color bar at the lower right. Orange dots show the fold change of the content of compounds in non-senescing and senescing leaves

The transmission of the BR signal from BRI1 located on the membrane to the transcription factor BZR1 in the nucleus is accomplished through a series of protein phosphorylation and dephosphorylation reactions. This series of phosphatases or phosphokinases, including BAK1, BSK, BIN2, and BZR1/2, are down-regulated in senescing leaves (Fig. 8).

### Gibberellin

**Fig. 9:**
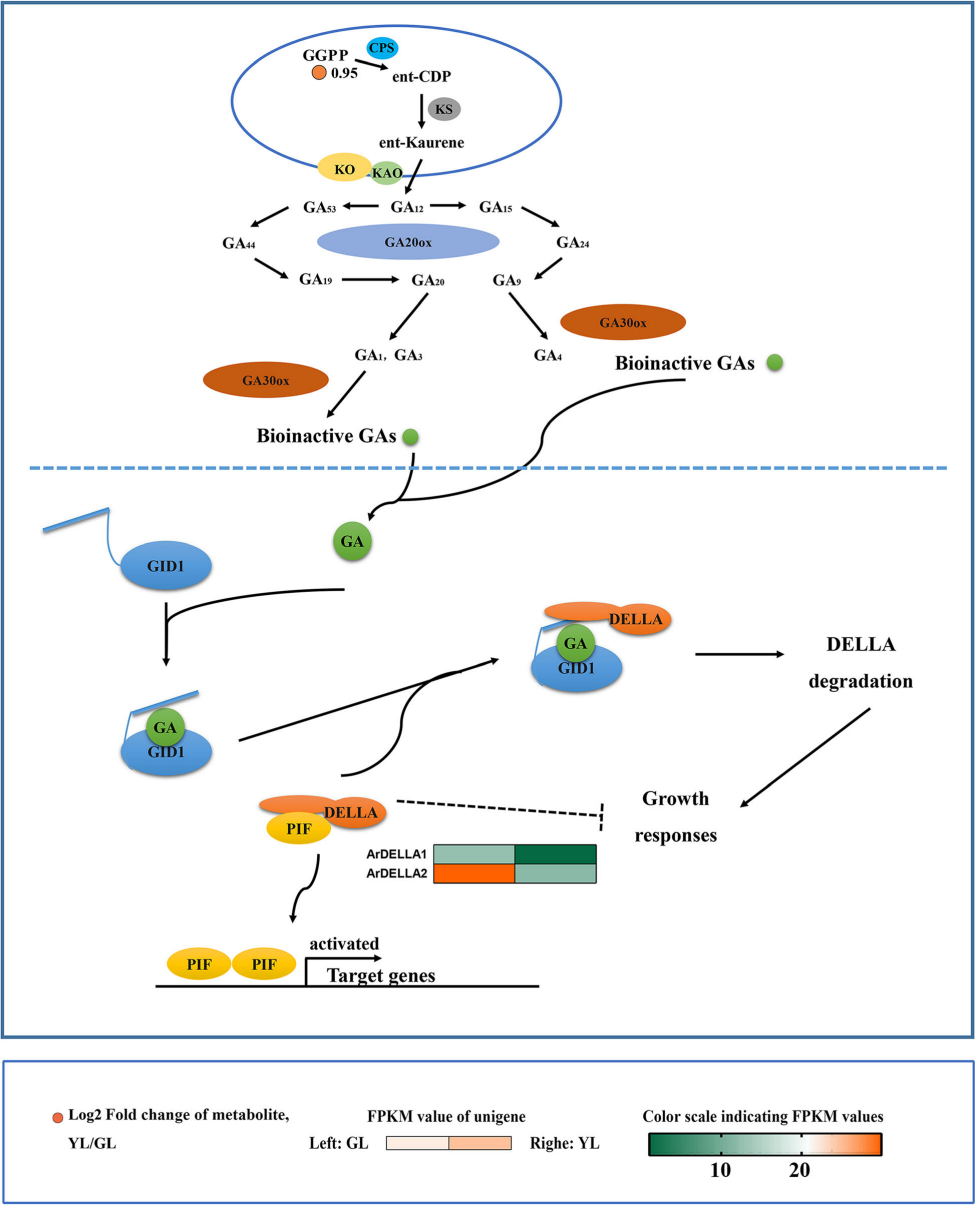
Biosynthetic and signal transduction pathways of gibberellins. GGPP, geranylgeranyldiphosphate; CPS, *ent*-copalyl diphosphate synthase; *ent*-CDP, *ent*-Copalyl diphosphate; KS, *ent*-kaurene synthase; KO, *ent*-Kaurene oxidase; KAO, *ent*-Kaurenoicacid oxidase; GA, Gibberellin; GA20ox, GA20-oxidase; GA30ox, GA30-oxidase; GID1, Gibberellin insensitive dwarf 1. GL, non-senescing leaves, fully expanded green leaves; YL, senescing leaves, completely yellow; ~ 95% chlorophyll loss. Colored cells represent the expression profiles of differentially expressed genes (DEGs). Heatmaps were generated using the Rpackage and the color bar at the lower right. Orange dots show the fold change of the content of compounds in non-senescing and senescing leaves

Gibberellin (GAs) are a class of phytohormones that belongs to the biguanide compounds, which play an important role in the entire life cycle of plants. The precursor of GA biosynthesis in higher plants is geranylgeranyldiphosphate (GGPP), which is synthesized from isopentenyl pyrophosphate. The impeding effect of gibberellin on leaf senescence has been discussed in many previous studies [72]. Senesent leaves have double the amout of GGPP than non-senescent (GL/YL = 0.5; Fig. 9). Of all the genes involved in the gibberellin signaling, only two differentially expressed genes (*ArDELLA1* and *ArDELLA2*) were found, all of which were highly expressed in non-senescing leaves.

## Discussion

### The promotion of leaf senescence in red maple

**Fig. 10:**
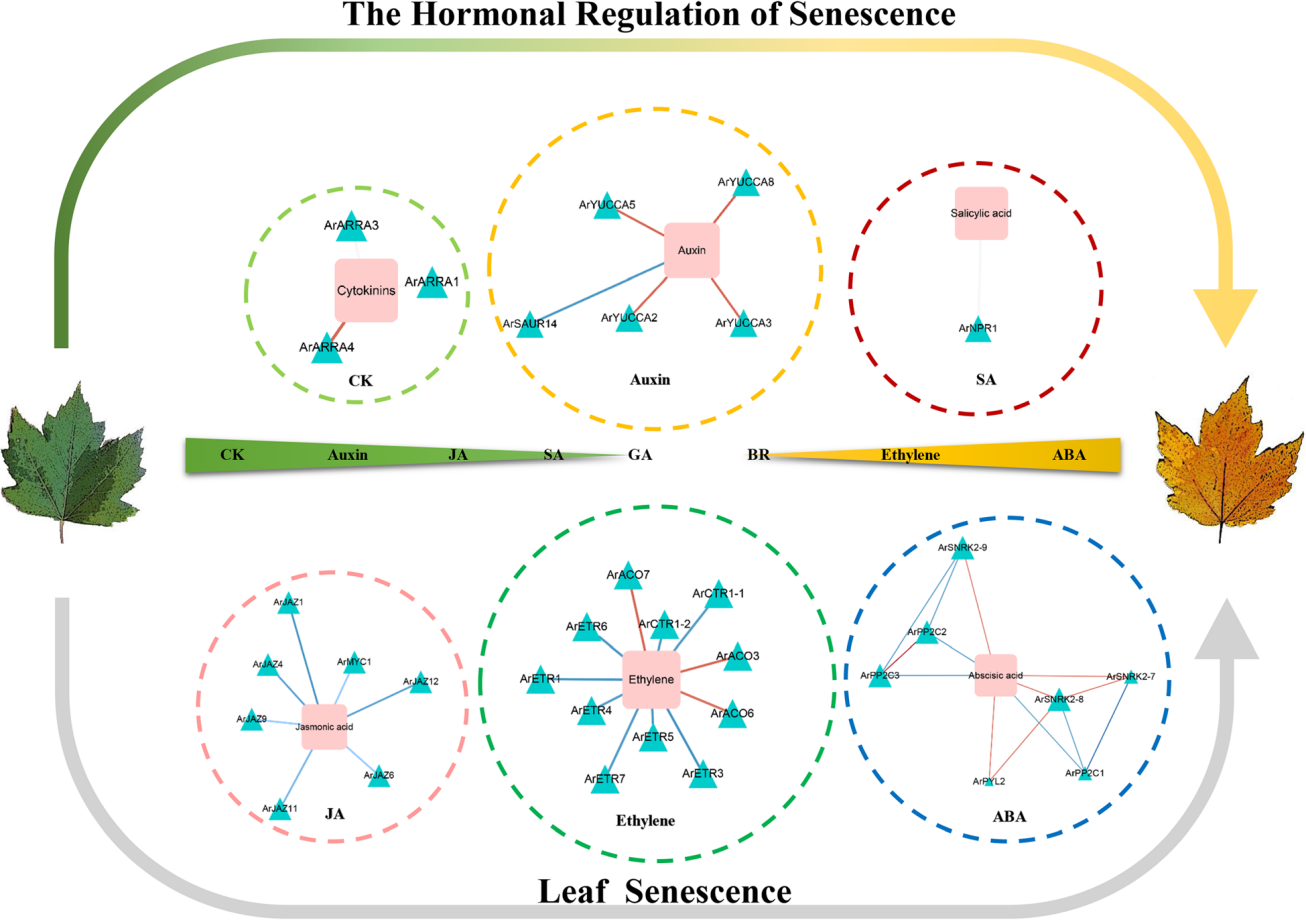
Gene regulatory network of leaf senescence with integrated data from red maple. Squares represent hormone-related metabolites and triangles represent regulatory genes. Two colored lines represent two different regulatory relationships between the regulatory genes and plant hormone-related metabolites; red refers to a positive regulatory relationship, blue refers to a negative regulatory relationship. CK, Cytokinins; JA, Jasmonic acid; SA, Salicylic acid; GA, Gibberellin; BR, Brassinosteroid; ABA, Abscisic Acid

Ethylene is a central regulator of growth and physiology in plants. From seed germination to organ senescence, and from sex determination to cell elongation, ethylene plays critical roles [73]. In some species, ethylene has been shown to be effective in the regulation of leaf senescence [74]. In this study, we observed a positive correlation between the levels of three ACC oxidases (*ArACO3*, *ArACO6*, and *ArACO7*) and ethylene in red maple (Fig. 10). The expression level of three *ArACOs* in senescing leaves was higher than in non-senescent and consistent with the level of ethylene. Early studies have demonstrated that ACC oxidase catalyzed the final step in ethylene biosynthesis, and under conditions of high ethylene production, ACO is often the rate-limiting step in biosynthesis [75, 76]. A study indicated that leaf senescence, as assessed by color change from green to yellow, was clearly delayed by 10–14 days in transgenic ACC oxidase antisense tomato plants when compared with wild-type plants [77]. ET signal transduction is performed according to the “linear” approach. It starts with a receptor and end with transcription factors (TFs). ETR (ethylene response) and CTR1 (Raf-like CONSTITUTIVE TRIPLE-RESPONSE1) are the negative regulatory factors of this pathway. For this study, two CTR1 genes (*ArCTR1–1* and *ArCTR1–2*) and six ETR genes (*ArETR1* and *ArETR3/4/5/6/7*) were negatively correlated with ethylene in red maple. Analyses of the *etr* mutants in Arabidopsis and rice, respectively, did indicate that *etr* leaves (ethylene insensitivity) had greater longevity than did wild-type leaves [78, 79]. Similarly, in *Arabidopsis thaliana* the *ctr1* mutation was analyzed in detail to show that the mutation causes a delay in functional leaf senescence [80]. These results suggested that the *ArACOs*, *ArETRs*, and *ArCTRs* were involved in the leaf senescence affected by ethylene in red maple.

It has been widely proven that ABA has a significant role in promoting leaf senescence [81]. Endogenous ABA levels can induce the expression of a variety of genes in various plants. Besides transcription factors and other signaling molecules, the products of these genes are functional genes such as LEA proteins, detoxification enzymes, and enzymes for osmoprotectant synthesis [82]. During the *Arabidopsis* senescence, most of the genes involved in the synthesis of abscisic acid were up-regulated [83–86]. In many circumstances, changes in product accumulation were consistent with gene expression. Interestingly, this presented the opposite results to our study. The ABA response has since been shown to be strictly controlled by intracellular signal transduction pathways, and many likely signaling intermediates correlated with ABA responses have also been identified by molecular and biochemical studies. Recent progress toward understanding early ABA signal transduction has led to the construction of a PYL–PP2C–SnRK2 signal transduction model [87]. A co-regulation network of ABA metabolic pathways in red maple showed that seven members of three gene families had correlations with ABA (Fig. 10). There was a negative correlation between the expression levels of *ArPP2C1/2/3* and ABA. *ArPYL2* and *ArSnRK2–7/8/9* were shown to be positively correlated with ABA levels. Numerous genetic evidences suggested that phosphatases 2C (PP2C) were negative ABA signaling regulators. Zhang et al. noted that the SAG113 gene in Arabidopsis encoded a protein phosphatase 2C, and the role of SAG113 protein phosphatase 2C was to inhibit stomatal closure during leaf senescence. This allowed increased oxygen to enter the leaves to speed up the respiration rate of the mesophyll cells, and the respiration rates of aging cells tended to accelerate, while simultaneously promoting the rapid evaporation of the leaf water until it was depleted [81, 88]. Our results indicated that four *ArPP2C* genes were highly expressed in senescing leaves, which suggested an important role for these genes in the leaf senescence of red maple. Previous research has shown that PYR/PYLs are ABA receptors functioning at the apex of a negative regulatory pathway that controls ABA signaling by inhibiting PP2Cs. Consequently, we speculated that in the senescing leaves of red maple, *ArPYL2* might interact with *ArPP2C1/2/3* and inhibit phosphatase activity, allowing *ArSnRK2–7/8/9* activation and the phosphorylation of target proteins, to then regulate leaf senescence.

### The inhibition of leaf senescence in red maple

Previous research suggests that cytokinins are one of the most crucial phytohormones in the regulation of plant growth and development [89, 90]. Cytokinins are likely the most commonly studied hormone that regulates leaf senescence. The application of exogenous cytokinin on detached plant leaves, can retard leaf senescence [89, 91]. The overexpression of components in the cytokinin signaling pathway may also delay plant leaf senescence [92]. In red maple, the content of cytokinin in non-senescing leaves is high. It has been suggested that cytokinin may inhibit leaf senescence in red maple. Correlation analysis showed that the content of cytokinins in red maple was positively correlated with ArARR-A1/3/4 (Fig. 10). It is known that the expression of type-A ARR genes is rapidly and specifically induced by cytokinins [93, 94]. This type of gene is considered to be a marker gene in the cytokinin signaling pathway. The overexpression of A-type ARR genes will lead to an earlier flowering time, longer roots, increased lateral roots, premature senescence, and reduced cytokinin sensitivity in transgenic plants. ARR16 in Arabidopsis may be involved in regulating the senescence process of its leaves [95]. In conclusion, our results suggested that ArARR-A1/3/4 in red maple may be involved in regulating the senescence process of leaves through the cytokinin signaling pathway.

Auxin plays an important role in all parts and stages of plant growth and development [96]. Early studies have shown that the external application of auxin had a negative correlation with leaf senescence, which suggests a role of auxin on the delay of leaf senescence [97]. In most cases, the application of auxin can delay both chlorophyll degradation and protein degradation in leaf disks for many plants, while the level of endogenous auxin were decreased in leaves that began/were in the process of senescing [54]. In this study, non-senescing red maple leaves were shown to contain elevated auxin levels in contrast to senescing leaves. Correlation analysis revealed that the auxin content of red maple was positively correlated with *ArYUCCA2/3/5/8* (Fig. 10). Previous studies have shown that yuc6-1d, a YUCCA6 activation mutant, had a high level of free IAA and displayed typical high-auxin phenotypes. The transgenic plants showed a significant life cycle extension and exhibited a significant inhibitory effect on dark- and hormone-induced senescence [98]. In addition, the expression of *ArSAUR14* was observed to increase during red maple senescence, which exhibited a negative regulatory relationship with auxin content. Recently, several molecular genetic studies found that the small auxin-up RNA (SAUR) gene, a member of the auxin-responsive gene family, played a critical role in plant growth by regulating the accumulation of the auxin phytohormone [R]. Transgenic rice plants overexpressing SAUR39 showed that this gene was expressed at higher levels in senescing leaves, unlike auxin biosynthesis. The transgenic plants had a lower auxin level and reduced polar auxin transport, as well as the down-regulation of some putative auxin biosynthesis and transporter genes [99]. Overall, the results suggested that ArYUCCA2/3/5/8 and ArSAUR14 in red maple may regulate leaf senescence through auxin metabolism and signaling.

### Two plant hormones in red maple that are contrary to previous studies

Jasmonates (JAs) and their oxylipin derivatives are well recognized phytohormones that regulate plant growth and plant adaptations to biotic stresses, as well as abiotic stresses [100]. The first confirmed biological function of JA was found as a senescence-promoting substance to promote the senescence of detached oat leaves [60]. He et al. (2002) showed that the JA content in senescing leaves was four-fold higher than that in non-senescing leaves [101]. However, our test results indicated that the JA content in the non-senescing leaves of red maple was higher than in senescing leaves. The detection results of JA downstream products revealed that the content of the MeJA in senescing leaves was moderately higher than in non-senescing leaves (GL/YL = 0.9). It is possible that JA generated additional MeJA to participate in the anti-stress reaction. The results of correlation analysis showed that the *ArJAZ1/4/6/9/11/12*, *ArMYC1*, and JA content were negatively correlated (Fig. 10). Previous studies have shown that JAZs can inhibit JA-induced leaf senescence by interacting with a series of transcription factors or signal transduction proteins such as MYC2 [102–105]. Therefore, the high expression of ArJAZs transcripts in senescing leaves of red maple may inhibit leaf senescence induced by JA.

Although SA has been well studied as a signaling molecule in plant disease defense responses, its role in leaf senescence has been recognized until recently. In Arabidopsis, the content of endogenous SA increased with the leaf senescence process [106], and the leaves of Arabidopsis mutants showed early or late senescence with increased or decreased SA content [107–112], respectively. We have shown that although the levels of phenylalanine dehydrogenase in senescing leaves is high, the content of salicylic acidis higher in non-senescent than senescent leaves. In many plants, salicylic acid is synthesized via the phenylalanine pathway [113]. Under the action of phenylalanine dehydrogenase, phenylalanine produces cinnamic acid, and then benzoic acid, and finally salicylic acid. However, cinnamic acid is the precursor of many polyphenols, such as flavonoids [114]. Therefore, we speculated that cinnamic acid in red maple is more involved in plant metabolism as a precursor of this type of substance. Correlation analysis revealed that there was a positive correlation between the ArNPR1 content and SA (Fig. 10). Previous studies confirmed that NPR1 plays an important role in leaf senescence. *npr1*, which is a mutant of SA signal receptor loss in Arabidopsis, exhibited the phenomenon of delaying leaf senescence and also changed expression of some Senescence-Associated Gene (SAGs) [106]. Consequently, we speculated that the lower accumulation of SA in senescing leaves might be related to the lower expression of the *ArNPR1* genes. Of course, this conjecture needs to be verified by further experiments in the future.

### A role of brassinosteroids and GA in leaf senescence of red maple

Brassinosteroids are a unique class of plant hormones that are essential for normal plant growth [115, 116]. However, to date, the roles of these substances in plant leaf senescence have not been studied in depth. At present, it is known that the seedling leaves of mung bean [117] and cucumber cotyledons [118] will appear to prematurely senesce when treated with epibrassinolide (*epiBR*). In Arabidopsis, different BR mutants (e.g., *det2*, *bri1*) showed the phenotype of accelerating or delaying leaf senescence [67–69]. In our results, the content of BRs in red maple was not detected, which may have been because the content of BRs varies significantly between different organisms. Previous studies revealed that the BRs content in pollen, immature seeds, and fruits was highest, followed by young growing tissues, whereas the BRS concentration in mature leaves was lowest [119, 120]. No differentially expressed genes were identified in the BRs synthesis pathway of red maple, and the genes of the signal transduction pathway were also highly expressed in non-senescing leaves without suggesting that there may be no significant accumulation of brassinosteroid during the senescence process.

Gibberellin is a type of plant hormone that belongs to diterpenoids, which plays an critical role over the entire life cycle of plants [121]. Based on the treatment of gibberellin and related research results, gibberellin was reported to maintain chlorophyll levels and RNA synthesis; thus, delaying post mitotic leaf senescence [R]. The spraying of exogenous GA (3) on Paris polyphylla can obviously inhibit the senescence of aerial components [122], and GA (4) can delay the senescence of *Alstroemeria hybrida* leaves [123, 124]. According to our results, no gibberellin was detected in red maple, and no differentially expressed genes were observed in the gibberellin synthesis pathway. Although the content of the initial GGPP metabolite in senescing leaves was high, the change of GA content during the senescing process of red maple could not be predicted. This is because the GGPP in plants can form GA, ent-kaurene [125], Chl-phytol (Chl (Phy)) [126–129], phytoene, and carotenoid [130] according to different branching pathways. The manipulation of any branching pathway using GGPP as the precursor, will also significantly affect other branching paths. For example, the up- or down-regulation of phytoene synthetase will lead to the increase or decrease of carotenoid synthesis and changes in GA levels [131]. Therefore, the effect of GA on leaf senescence of red maple requires further study.

## Conclusion

Ethylene and ABA are likely to play a positive role in the leaf senescence of *Acer rubrum*. The key factors involved in the ethylene regulation of leaf senescence may include members of the *ArACO*, *ArETR*, and *ArCTR* gene families. In the ABA signaling pathway, the PYL-PP2C-SnRK2 component plays a critical role in regulating ABA production. Cytokinins and auxins are likely to play negative roles in the regulation of leaf senescence of red maple. A-type nuclear response regulators are very likely to be key regulators of cytokinin in red maple to delay leaf senescence. It is suggested that future research on the mechanisms of auxin delaying leaf senescence of this species might focus on genes such as YUCCA. The higher accumulation of JA and SA in non-senescing leaves of red maple was contrary to the research results of others in that the content of endogenous JA and SA in other species increased with leaf senescence.

Based on the analytical results of the changes in the content of related metabolites of the JA synthesis pathway, we speculated that JA in the senescing leaves of red maple was involved a period of rapid accumulation. We speculated further, that the precursor of SA, cinnamic acid, was more involved with other secondary metabolites, such as flavonoids, during aging, which may have been the reason that SA was not further accumulated. We did not detect BRs and GAs in red maple. Based on the analysis of the expression of differential genes in the BRs signaling pathway, we speculated that it was unlikely that the content of this substance increased during the senescence of red maple leaves. Due to the complexity of the downstream branch pathway of the initial metabolite of the GA metabolic pathway, GGPP, we still cannot speculate on the possible accumulation of GAs. In the future, we will further elucidate the mode and mechanism of action of the above hormones in the regulation of senescing red maple leaves through additional experimental evidence.

## Supplementary information

**Additional file 1: Table S1.** Differentially expressed plant hormone-related metabolites candidates in red maple.

**Additional file 2: Table S2.** Differentially expressed gene (DEG) candidates involved in plant hormone metabolism in red maple.

**Additional file 3: Table S3.** Correlation matrix of metabolites (plant hormone-related) and gene expression levels.

**Additional file 4: Table S4.** Classification of macromolecules in senescing leaves compared with non-senescing leaves in red maple. “Total” refers to the total number of metabolites changed from senescent to non-senescent leaves.

**Additional file 5: Table S5.** Functional categories of genes correlated with the anabolism of macromolecules differentially expressed in senescing leaves compared with non-senescing leaves in red maple.

## Data Availability

All the sequencing data were submitted to NCBI Sequence Read Archive database under accession number PRJNA531583.

## Abbreviations

UHPLC-QE-MS: Liquid chromatograph Q extractive mass spectrometry
ET: Ethylene
ABA: Abscisic acid
JA: Jasmonic acid
SA: Salicylic acid
BR: Brassinosteroid
CK: Cytokinin
IAA: Auxin
GA: Gibberellin
Non-senescing leaves: Fully expanded green leaves
senescing leaves: 100% yellowing leaf surface, ~ 95% chlorophyll loss
IDA: Information-dependent acquisition
ESI +: Electrospray ionization-positive ion mode
ESI–: Electrospray ionization-negative ion mode
NGS: Next-generation sequencing
SMRT: Single-molecule real-time sequencing
Iso-Seq: Isoform Sequencing protocol
FPKM: Fragments per kilobase of transcript per million mapped reads
DEGs: Differentially expressed genes
NCBI: National Center for Biotechnology Information
GL: Non-senescing leaves, fully expanded green leaves
YL: Senescing leaves, 100% yellowing of the leaf surface; ~ 95% chlorophyll loss
mtnK: 5-methylthioribose kinase
mtnA: Methylthioribose-1-phosphate isomerase
MTN: 5′-methylthioadenosine nucleosidase
mtnB: Methylthioribulose-1-phosphate dehydratase
speE: Spermidine synthase
mtnC: 2,3-diketo-5-methylthiopentyl-1-phosphate enolase-phosphatase
speD: S-adenosylmethionine decarboxylase proenzyme
mtnD: 1,2-dihydroxy-3-keto-5-methylthiopentene dioxygenase
metK: S-adenosylmethionine synthetase
TAT: Tyrosine aminotransferase
ACS: 1-aminocyclopropane-1-carboxylate synthase
ACO: Aminocyclopropanecarboxylate oxidase
ETR: Ethylene receptor
CTR: Constitutive
EIN2: Ethylene insensitive 2
EIN3: Ethylene insensitive 3
ERF1/2: Ethylene-responsive transcription factor 1/2
TF: Transcription factor
ZEP: Zeaxanthin epoxidase
NSY: Neoxanthin synthase
VDE: Violaxanthin de-epoxidase
ABA: Xanthoxin dehydrogenase
NCED: 9-cis-epoxycarotenoid dioxygenase
AAO: Abscisic-aldehyde oxidase
PYL: Pyrabactin-resistance 1-like
PP2C: Protein phosphatase type 2C
SnRK: Serine/threonine-protein kinase
ABF: Abscisic acid responsive element binding factor
DMAPP: Dimethylallyl diphosphate
ATP: Adenosine triphosphate
ADP: Adenosine diphosphate
AMP: Adenosine monophosphate
IPT: Isopentenyl transferase
iPRTP: Isopentenyl adenosine-5′-triphosphate
iPRDP: Isopentenyl adenosine-5′-diphosphate
iPRMP: Isopentenyl adenosine-5′-monophosphate
CYP735A: Cytokinin trans-hydroxylase
tZRTP: Trans-Zeatin riboside triphosphate
tZRDP: Trans-Zeatin riboside diphosphate
tZRMP: Trans-Zeatin riboside monophosphate
LOG: Cytokinin-activating enzyme LONELY GUY
iPR: Isopentenyl adenosine
tZR: Trans-zeatin riboside
iP: Isopentenyl adenine
tZT: Trans-zeatin
AHP: Histidine-containing phosphotransfer peotein
Type-A ARRs: Type-A response regulators
Type-B ARRs: Type-B response regulators
trpB: Tryptophan synthase beta chain
Trp: Tryptophan
DDC: Tryptophan decarboxylase
TAA: Tryptophan aminotransferase
TAM: Tryptamine
IPA: Indole-3-pyruvate
CYP71A13: Indoleacetaldoxime dehydratase
IAOx: Indole 3-acetaldoxime
YUCCA: Indole-3-pyruvate monooxygenase
IAAld: Indole-3-acetaldehyde
IAN: Indole-3-acetonitrile
ALDH: Aldehyde dehydrogenase
NIT: Nitrilase
IAA: Indole-3-acetate
AUX/IAA: Auxin-responsive protein IAA
ARF: Auxin response factor
TIR1: Transport inhibitor response 1
SAUR: Small auxin up-regulated RNA
LA: Phospholipase A1
α-LeA: Alpha-Linolenic acid
LOX: Lipoxygenase
12,13-HPOT: 12,13-hydroperoxylinoleic acid
AOS: Allene oxide synthase
12,13-EOT: 12,13-epoxyoctadecatrienoic acid
AOC: Allene oxide cyclase
OPDA: Oxophytodienoic acid
OPR: Oxophytodienoic acid reductase
JMT: Jasmonic acid carboxyl methyltransferase
JAR1: Jasmonic acid resistant 1
MeJA: Methyl jasmonate
JA-Ile: Jasmonoyl-L-isoleucine
COI1: Coronatine-insensitive protein 1
JAZ: Jasmonate ZIM domain-containing protein
PAL: Phenylalanine ammonia-lyase
BA2H: Benzoate
NADPH: Oxygen oxidoreductase (2-hydroxylating)
NPR: Nonexpressorofpathogenesis-relatedgenes1
TGA: Transcription factor TGA
90B/724B: Steroid 22-alpha-hydroxylase
6-DeoxoCT: 6-Deoxocathasterone
6-OHCN: 6alpha-Hydroxycampestanol
6-DeoxoTE: 6-Deoxoteasterone
6-OxoCN: 6-Oxocampestanol
6-Deoxo3DT: 3-Dehydro-6-deoxoteasterone
CT: Cathasterone
6-DeoxoTY: 6-Deoxotyphasterol
TE: Teasterone
6-DeoxoCS: 6-Deoxocastasterone
3-DT: 3-Dehydroteasterone
6-OHCS: 6alpha-Hydroxy-castasterone
TY: Typhasterol
85A1/2: Brassinosteroid-6-oxidase 1
92A6: Typhasterol/6-deoxotyphasterol 2alpha-hydroxylase
85A2: Brassinosteroid-6-oxidase 2
BRI1: Protein brassinosteroid insensitive 1
BAK1: Brassinosteroid insensitive 1-associated receptor kinase 1
BSK: BR-signaling kinase
BSU1: Serine/threonine-protein phosphatase
BIN2: Brassinosteroid insensitive 2
BZR1/2: Brassinosteroid resistant 1/2
PP2A: Protein phosphatase type 2A
GGPP: Geranylgeranyldiphosphate
CPS: Ent-copalyl diphosphate synthase
ent-CDP: Ent-Copalyl diphosphate
KS: Ent-kaurene synthase
KO: Ent-Kaurene oxidase
KAO: Ent-Kaurenoicacid oxidase
GA: Gibberellin
GA20ox: GA20-oxidase
GA30ox: GA30-oxidase
GID1: Gibberellin insensitive dwarf 1
